# Exploring the Drug-Loading and Release Ability of FucoPol Hydrogel Membranes

**DOI:** 10.3390/ijms241914591

**Published:** 2023-09-26

**Authors:** Diana Araújo, Matilde Martins, Filomena Freitas

**Affiliations:** 1 Associate Laboratory i4HB—Institute for Health and Bioeconomy, School of Science and Technology, NOVA University Lisbon, 2829-516 Caparica, Portugal; df.araujo@campus.fct.unl.pt (D.A.); mt.martins@campus.fct.unl.pt (M.M.); 2 UCIBIO—Applied Molecular Biosciences Unit, Department of Chemistry, School of Science and Technology, NOVA University Lisbon, 2829-516 Caparica, Portugal

**Keywords:** hydrogel membranes, FucoPol, loading methods, caffeine, diclofenac sodium

## Abstract

The polysaccharide FucoPol has recently been shown to yield hydrogel membranes (HMs) characterized by good mechanical properties, biocompatibility, and anti-inflammatory activity that render them promising biomaterials for use in the biomedical field. Subsequently to such findings, envisaging their development into novel delivery systems for topical applications, in this study, FucoPol HMs prepared by crosslinking the biopolymer with iron cations were loaded with caffeine or diclofenac sodium as model drugs. Two loading methods, namely diffusion and mixing, were applied to evaluate the FucoPol’s HM drug-loading capacity and entrapment efficiency. The diffusion method led to a higher caffeine loading (101.9 ± 19.1 mg/g) in the HM1_D_CAF_ membranes, while the mixing method resulted in a higher diclofenac sodium loading (82.3 ± 5.1 mg/g) in the HM1_D_DS_ membranes. The HM1_D_CAF_ membranes were characterized by increased mechanical and rheological parameters, such as their hardness (130.0 ± 5.3 kPa) and storage modulus (1014.9 ± 109.7 Pa), compared to the HM1_D_DS_ membranes that exhibited lower values (7.3 ± 1.2 kPa and 19.8 ± 3.8 Pa, respectively), probably due to leaching occurring during the drug-loading process. The release profiles revealed a fast release of both APIs from the membranes loaded by diffusion, while a prolonged and sustained release was obtained from the membranes loaded by mixing. Moreover, for all API-loaded membranes, the release mechanism followed Fickian diffusion, with the release rate being essentially governed by the diffusion process. These findings, together with their previously shown biological properties, support the suitability of the developed FucoPol HMs to be used as platforms for the topical delivery of drugs.

## 1. Introduction

Hydrogel membranes (HMs) are hydrated porous media constructed from hydrophilic polymers crosslinked to three-dimensional network structures [1,2] that possess unique properties, namely high water content, flexibility, and good mechanical strength. Such features make HMs suitable for use in topical and transdermal applications, including drug-delivery systems [2] and wound dressings [3]. HM use as transdermal drug-delivery systems is supported by their capacity to protect the drugs in hostile environments (e.g., enzyme degradation and low pH) and promote the release of the loaded drugs in desired targeted sites [4,5].

Several approaches have been reported for the incorporation of active pharmaceutical ingredients (APIs) within the HM structure, including permeation/diffusion, entrapment/mixing, and covalent bonding [6,7]. Diffusion is a simple and easy method that involves adsorption of the drug onto the membrane structure and it is achieved by soaking the pre-formed membrane in a drug-containing solution. The drug diffusion into the gel matrix is determined by several factors, such as the structure porosity, the size of the drug, and its chemical nature. This approach is only suitable for loading small molecules that can easily migrate through the small pores of the hydrogel. It allows for high loading efficiencies of hydrophilic drugs and decreases the possibility of drug deactivation [7,8]. The mixing method, on the other hand, consists of physically trapping the drug within the hydrogel matrix by mixing it with the polymer solution before gelation. This method allows the loading of larger and hydrophobic drugs and increases the duration of release time. However, in addition to increasing the chance of drug deactivation, it can also lead to unnecessary crosslinking [9,10].

Diclofenac sodium is a nonsteroidal anti-inflammatory drug (NSAID) commonly used for the treatment of pain and inflammation. Its properties, such as small molecular weight, lipophilic nature, and ability to penetrate the skin barrier, make it a good candidate for transdermal drug delivery [11]. Diclofenac sodium is sparingly water-soluble, but has good solubility in various organic solvents (e.g., ethanol, DMSO), making it easy to incorporate into HMs using diverse loading methods, including permeation [8,12] and entrapment [10,13]. Caffeine is a stimulant of the central nervous system, and it is widely used as a model hydrophilic compound for skin toxicology studies. Its topical application prevents excessive fat accumulation in the skin, supports lymphatic drainage, and protects the skin from photodamage [14,15]. Due to its properties, caffeine has been integrated into various topical or transdermal formulations for different applications, including scalp stimulation to promote hair growth [16] and antiaging and antioxidant agents in skincare creams [17].

FucoPol is a bacterial polysaccharide that was recently used as a structuring agent to fabricate HMs characterized by good mechanical properties [18]. The HMs were obtained by crosslinking FucoPol with Fe^3+^, resulting in flexible, porous structures with high swelling ability. Moreover, FucoPol HMs displayed anti-inflammatory activity, which renders them additional interest for exploitation in biomedical applications. In this study, FucoPol HM membranes were loaded with caffeine or diclofenac sodium as model drugs. The impact of the loading method used, diffusion or mixing, was evaluated, and the loaded HMs’ mechanical and rheological properties were characterized. Finally, their drug loading and release capacity was evaluated envisaging their future use as drug-delivery systems.

## 2. Results and Discussion

### 2.1. Loading of APIs

The ability of the HM1 membranes to be loaded with APIs was evaluated by a diffusion method, in which freeze-dried membrane samples were soaked in two individual APIs solution, namely caffeine, a stimulant of the central nervous system with a well-known effect on adipocyte lipolytic activity [15], and diclofenac sodium, an NSAID widely used in pain management [11]. To study the impact of the loading method, HM1 membranes were also loaded with APIs individually, following a mixing method where a specific amount of each API was mixed with the freeze-dried FucoPol and API loading occurred through drug entrapment during the gelation process.

**Figure 1:**
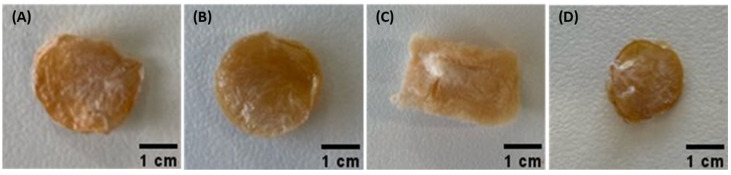
Macroscopic aspect of the freeze-dried FucoPol hydrogel membranes loaded with caffeine (**A**,**B**) prepared by diffusion (HM1_D_CAF_) or mixing (HM1_M_CAF_) methods, respectively, and loaded with diclofenac sodium (**C**,**D**) prepared by diffusion (HM1_D_DS_) or mixing (HM1_M_DS_) methods, respectively.

As shown in Figure 1, macroscopically, all the freeze-dried loaded membranes retained their brownish coloration, lightness, and fragility, while keeping their dimensions. However, the HM1_D_DS_ presented a rectangular shape and a lighter coloration. These changes in shape are related to the loss of consistency of the freeze-dried HM1 membranes during immersion in the diclofenac loading solution caused by the possible interaction of the API molecules with the Fe^3+^ ions.

The key parameters used to evaluate the loading ability of the HM1 membranes were the drug loading (DL) and the entrapment efficiency (EE), which represent the mass ratio of drug to drug-loaded HM and the efficacy of HM to retain the drug in its structure, respectively. As shown in Table 1, the diffusion method resulted in a higher DL capacity for caffeine (101.9 ± 19.1 mg/g) than for diclofenac sodium (53.9 ± 11.8 mg/g), which correlates with the API concentration in the loading solutions. The higher concentration of caffeine in the loading solution (1.0 wt.%) resulted in a higher amount of caffeine being loaded into the membrane matrix. On the other hand, owing to the lower water solubility of diclofenac sodium [19], a lower API concentration was used (0.1 wt.%), thus resulting in a lower DL. However, higher EE was noticed for diclofenac sodium (1.6 ± 0.2%) than for caffeine (0.6 ± 0.1%) (Table 1). Although these results might be significantly impacted by the API concentration in the drug-loading solution, the physicochemical characteristics of the APIs also contributed to this behavior. In fact, during the loading procedure, the negative ions of diclofenac might have interacted with the Fe^3+^ cations, probably promoting their leaching from the membrane structure and, consequently, it became less consistent. Moreover, the leaching process might have promoted the accommodation of water molecules inside the membrane structure, increasing their dimensions but decreasing their density. This is corroborated by the fact that after freeze-drying, the HM1_D_DS_ membranes were lighter and presented an altered shape, as observed in Figure 1. The impact of the API concentration in the loading solution on the drug-loading amount was also reported for other structures including the CGC hydrogels [20] and chitosan/β-glycerophosphate hydrogels [8]. For the same concentration of diclofenac loading solution (0.1 wt.%), the latter structures presented a lower DL value (2.48 mg/g) which might be explained by the low swelling ability of those hydrogels [8].

The low EE observed for loading both tested APIs by the diffusion method is correlated with the membrane swelling capacity, which was limited by their low porosity and tight microstructure [18]. Following a similar loading methodology, CGC-based hydrogels obtained higher and similar EE values for caffeine and diclofenac sodium (13.11 ± 0.49% and 14.70 ± 0.60%, respectively) [20]. This difference can probably be justified by the high porosity of CGC hydrogels (79.4 ± 0.60%) when compared to HM1 membranes (59.3 ± 8.3%) [18]. Additionally, the loading might also be influenced by the low drug-polymer interactions resulting from repulsion between the anionic groups of FucoPol and APIs molecules [21].

Significantly higher EE was obtained with the mixing method for loading both tested APIs into HM1 compared to the diffusion method (Table 1). Moreover, the EE values were similar for caffeine and diclofenac sodium (25.8 ± 3.5% and 25.4 ± 3.6%, respectively). This result indicates that an identical API amount was entrapped within the membrane structure and that the nature of the API had no significant impact. However, these values are low when compared to other structures and the explanation might be related to API leakage during the preparation process since the polymer solution containing the API was immersed in FeCl_3_ solution for crosslinking. Tan et al. [10] reported EE values of 56% and 65% for diclofenac sodium loading in carboxymethyl sago pulp/chitosan hydrogels. In another study, chitosan/PVA hydrogels were loaded with caffeine, and an EE of around 99% was reported [22]. The same amount of API (0.025 wt.%) was used for both caffeine and diclofenac sodium loading, resulting in a similar DL for diclofenac (82.3 ± 5.1 mg/g) and caffeine (78.6 ± 2.5 mg/g). These results suggest that the physicochemical characteristics of the APIs did not affect the loading process efficiency.

Overall, the results obtained showed that a higher DL for caffeine was achieved using the diffusion method while for diclofenac sodium the mixing method allowed the reaching of higher DL values. Furthermore, despite the lower concentration of API used, following the mixing method led to higher values of EE for both APIs. In general, it was demonstrated that drug loading can be affected by the methodology used to load the API as well as the physicochemical properties of the API used.

### 2.2. Characterization of the Loaded HM1

#### 2.2.1. Fourier Transform Infrared (FT-IR) Spectroscopy

**Figure 2:**
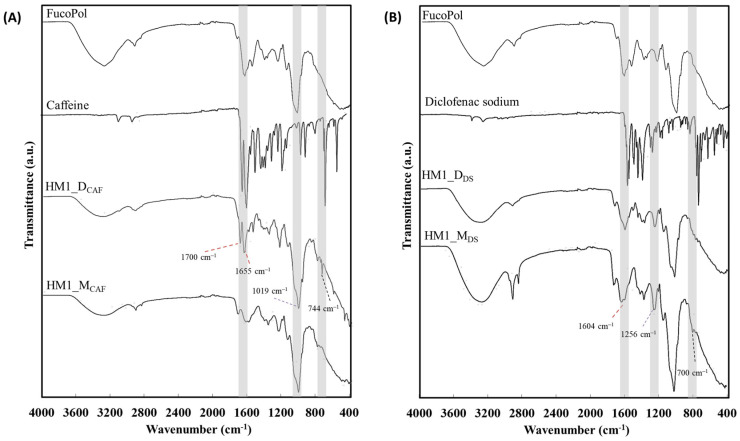
FT-IR spectra of HM1 loaded with (**A**) caffeine and (**B**) diclofenac sodium by diffusion (HM1_D) and mixing (HM1_M) methods.

The structural characterization of the loaded HM1 was carried out by FT-IR analysis. As shown in Figure 2, the loaded membranes exhibited a spectrum similar to that of FucoPol. However, the incorporation of the APIs within their structure led to some structural modifications. It can be observed that the spectra of the API-loaded membranes displayed the characteristic adsorption bands of FucoPol, along with specific vibrations of each API and the intensity of these vibrations is consistent with the API content. The spectrum of caffeine presents absorption peaks at 3115 and 2952 cm^−1^ (C-H stretching of methyl groups), 1697 and 1650 cm^−1^ (C=O stretching of amide I), and 1549 cm^−1^ (C=N stretching of amide II) [23] (Figure 2A). The HM1_D_CAF_ and HM1_M_CAF_ membranes exhibited a decrease in the intensity of the broadband of O-H stretching of hydroxyl groups at 3300 cm^−1^ and the C-H stretching at 2927 cm^−1^. Figure 2A shows that the incorporation of caffeine can be confirmed by the increasing intensity of the peaks attributed to the C=O stretching at around 1700 cm^−1^ and 1655 cm^−1^, and increased C-N and C-C vibrations at 1019 cm^−1^. Moreover, the presence of an additional peak at around 744 cm^−1^ confirms the presence of caffeine [24]. The low magnitude of these peaks in the spectra of the loaded membranes prepared by mixing corroborates the lower caffeine content in these structures (Table 1).

The diclofenac sodium spectrum comprised absorption peaks at 3387 cm^−1^ (N-H stretching), 1603 cm^−1^ (C=C ring skeletal vibration), 1573 cm^−1^ (COO- anti-symmetrical vibration), 1350–1250 cm^−1^ (C-N stretching) and 730–745 cm^−1^ (C-H out of plane, di and tri substituted rings) [25] (Figure 2B). Despite the low intensity of the peaks, the presence of diclofenac in the HM1_D_DS_ and HM1_M_DS_ membrane structures can be confirmed by the absorption peak at 1604 cm^−1^, the increased intensity of the peak at 1256 cm^−1^ and the appearance of small peaks around 700 cm^−1^ (Figure 2B).

When an API is loaded into a membrane structure, it may interact with the polymer matrix and affect several of its properties such as the mechanical strength and the viscoelastic properties.

#### 2.2.2. Mechanical Properties

**Figure 3:**
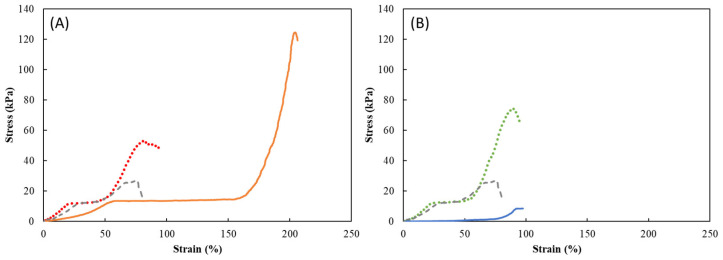
FTR spectra of HM1 loaded with (**A**) caffeine and (**B**) diclofenac sodium by diffusion (HM1_D) and mixing (HM1_M) methods. Compression stress–strain curves of the HM1 membranes (light grey dashed line) and the HM1 membranes loaded with (**A**) caffeine and (**B**) diclofenac sodium by diffusion (full lines, orange for caffeine and blue for diclofenac sodium) and mixing (dotted lines, red for caffeine and green for diclofenac sodium) methods.

The impact of dehydration/rehydration and the presence of the API on the mechanical properties of the HM1 membranes is shown in Figure 3. It can be noticed that loading caffeine into the HM1 membranes either by diffusion or mixing methods, led to significant changes in the mechanical parameters. The hardness values of the caffeine-loaded membranes (130.0 ± 5.3 kPa and 60.4 ± 7.2 kPa, respectively) were considerably higher than those displayed by the original HM1 membranes (32.4 ± 5.8 kPa) (Table 2). Moreover, the HM1_D_CAF_ membranes presented a higher ability to withstand the deformation, since their rupture only occurred at 228.8% of strain (Figure 3A). This result might be explained by the effect of the freeze-drying process and the incorporation of the caffeine that reinforced the membrane structure [26]. Consequently, the HM1_D_CAF_ and HM1_M_CAF_ membranes presented higher compressive modulus (34.1 ± 5.2 kPa and 66.5 ± 8.5 kPa, respectively) and toughness (41.2 ± 0.6 kJ/m^3^ and 16.3 ± 0.5 kJ/m^3^, respectively). A similar trend was reported for CGC hydrogels, for which loading caffeine into the hydrogels resulted in higher hardness (15.6 ± 2.53 kPa), compressive modulus (120.0 ± 61.64 kPa), and toughness (120.0 ± 61.64 kJ/m^3^) than the original ones (5.04 ± 0.14 kPa, 23.0 ± 0.89 kPa, and 0.78 ± 0.01 kJ/m^3^, respectively) [27]. Chee et al. [26] also described changes in some mechanical parameters, such as a decrease in tensile strains and higher Young’s modulus values of polyvinyl alcohol hydrogels when loaded with caffeine. Although there are other factors to consider such as the number of freeze-thaw cycles and the polymer orientation, the authors assigned this effect to the caffeine crystallization during the drying process.

The HM1_M_CAF_ membranes displayed lower values of hardness (60.4 ± 7.2 kPa), and toughness (16.3 ± 0.5 kJ/m^3^) when compared to those loaded by the diffusion method (130.0 ± 5.3 kPa, 41.2 ± 0.6 kJ/m^3^, respectively), which can be justified by the higher amount of caffeine present in the latter structures (Table 1). Similarly, Yang et al. [28] showed that increasing the methacrylamide dopamine content from 3% to 9% in chitosan-based hydrogels enhanced the compressive stress from 19 to 37 kPa and those values were higher than the ones displayed by the non-loaded hydrogel (18 kPa).

The results obtained for the HM1_D_DS_ and HM1_M_DS_ membranes are displayed in Figure 3B. Contrary to that obtained for caffeine, the loading method used to incorporate diclofenac within the membrane structure had a very significant impact on their mechanical parameters. It can be observed that the HM1_M_DS_ membranes exhibited higher hardness (81.8 ± 3.4 kPa), compressive modulus (67.7 ± 2.5 kPa), and toughness values (19.7 ± 0.6 kJ/m^3^) than those loaded by the diffusion method (7.3 ± 1.2 kPa, 4.6 ± 1.3 kPa, and 1.7 ± 0.4 kJ/m^3^, respectively) (Table 2). Despite the lower amount of diclofenac present in these later structures, the leaching phenomenon has probably contributed to the observed fragility of the membranes (Figure 1), and consequently, a decline in the mechanical properties. Wong et al. [29] evaluated the effect of loading polyethylene oxide (PEO) hydrogel films with diclofenac sodium via diffusion and mixing methods. The authors described a decrease in the mechanical properties of the hydrogel films and attributed it to a significant decrease in polymer crystallinity for the diffusion method and a low crosslinking density for the mixing method.

#### 2.2.3. Rheological Properties

**Figure 4:**
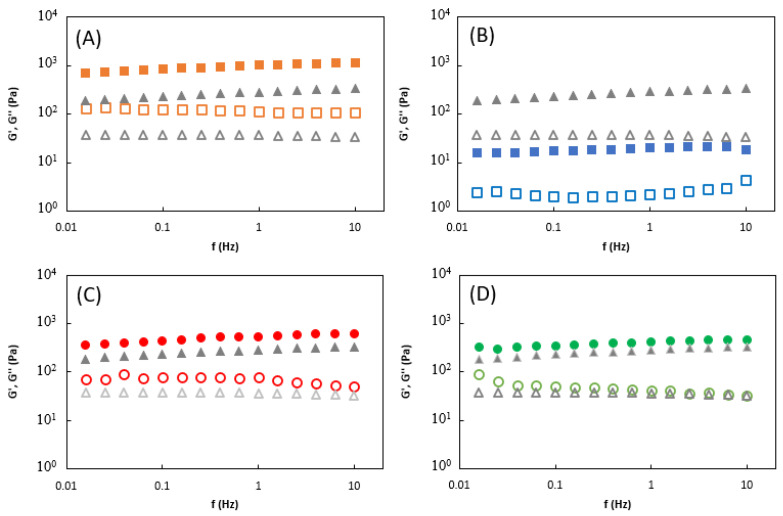
Rheological properties of the HM1 membranes (triangle grey symbols) and the HM1 membranes loaded with caffeine using (**A**) diffusion and (**C**) mixing method, and with diclofenac sodium using (**B**) diffusion and (**D**) mixing method, at 25 °C. Diffusion and mixing methods are represented by square and circle symbols, respectively. Mechanical spectrum storage (G′, solid symbols) and loss moduli (G″, open symbols).

The effect of dehydration/rehydration and the presence of the API on the viscoelastic properties of the HM1 membranes is shown in Figure 4. In general, all loaded membranes presented a rheological profile identical to the original HM1 membranes with a predominant elastic behavior, suggesting that the incorporation of APIs had no significant impact on the viscoelastic properties of the structure. As solid-like structures, the values of the storage modulus were one order of magnitude higher than the loss modulus values over almost the entire range of frequency. Similar behavior was reported for other ion-crosslinked hydrogels, namely hydrogels based on nanofibrillated cellulose [30], succinoglycan [31], and gellan [32]. As shown in Figure 4A,C, the HM1_D_CAF_ and HM1_M_CAF_ membranes presented higher storage and loss moduli which is consistent with the observed enhancement of the structures’ mechanical properties (Figure 3A). As demonstrated in Table 2, at a frequency of 1 Hz, the G′ value was enhanced from 285.8 ± 36.1 Pa to 1014.9 ± 109.7 (Figure 4A) and 609.7 ± 78.3 Pa (Figure 4A), while the G″ increased from 36.7 ± 5.5 to 113.2 ± 13.7 and 80.3 ± 2.7 Pa, for the HM1_D_CAF_ and HM1_M_CAF_ membranes, respectively. These results demonstrate that loading caffeine into the membranes can potentially impact the crosslinking density, affecting their ability to store and recover elastic energy. Moreover, the addition of caffeine to membranes may alter the molecular mobility and the relaxation behavior of the polymer chains which can affect internal friction and energy dissipation within the hydrogel network [33]. A similar improvement of rheological parameters was reported for the CGC hydrogel loaded with caffeine by diffusion [27]. In that study, at a frequency of 1 Hz, the caffeine-loaded hydrogels presented G′ and G″ values of 315.0 ± 76.7 Pa and 29.3 ± 8.4 Pa, respectively, while lower values were obtained for the original hydrogels (149.9 ± 9.8 Pa and 11.9 ± 0.5 Pa, respectively).

The rheological properties of the HM1_D_DS_ and HM1_M_DS_ membranes showed that the loading method also had an impact on the storage and loss moduli values compared to the original HM1 membranes (Figure 4B,D). The most relevant difference was obtained for the HM1_D_DS_ membranes on which, at a frequency of 1 Hz, the G′ and G″ values were drastically reduced from 285.8 ± 36.1 Pa and 36.7 ± 5.5 Pa to 19.8 ± 3.8 Pa and 2.2 ± 0.1 Pa, respectively (Figure 4B). Following a similar loading method, the incorporation of diclofenac sodium within the structure of starch/pectin hydrogels led to a decrease in the rheological parameters compared to the original hydrogels, namely for the G′ values that decreased from 353.36 Pa to 72.74 Pa, suggesting that the interaction between the diclofenac and the carboxyl groups of the polymers prevent the original hydrogen bond formation among them [34]. On the other hand, performing the loading by mixing, an increase in the G′ value from 285.8 ± 36.1 Pa to 421.0 ± 107.3 Pa was observed and a similar value of G″ was obtained (40.6 ± 10.2 Pa), at a frequency of 1 Hz (Table 2). The results suggest that mixing diclofenac with the polymer before gelation may promote the formation of additional crosslinking between the hydrogel matrix and the drug molecules. Russo et al. [35] also reported a slight increase in G′ values (21.61 ± 1.45 kPa) of diclofenac-loaded poloxamer gels when compared to non-loaded gels (18.58 ± 0.50 kPa).

Overall, these findings suggest that the addition of APIs to the membranes has an impact on their mechanical and rheological properties, which should be considered for designing drug-delivery systems.

### 2.3. In Vitro Release Studies

**Figure 5:**
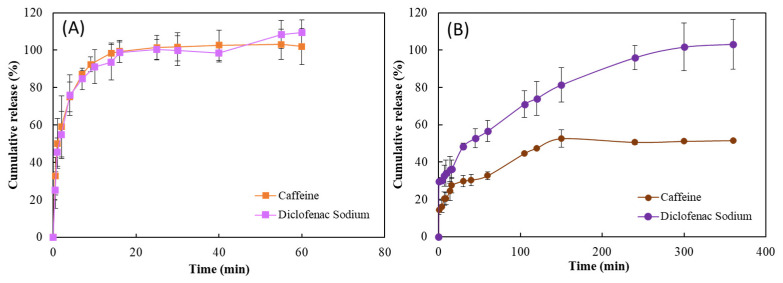
Cumulative release profile of membranes (**A**) HM1_D_CAF_ (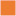) and HM1_D_DS_ (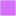) and (**B**) HM1_M_CAF_ (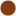) and HM1_M_DS_ (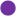), in deionized water, at 37 °C.

The release studies were performed by placing the API-loaded HM1 membranes in deionized water, at 37 °C, and evaluating the cumulative release profiles of the APIs from the membranes. As shown in Figure 5 different release profiles could be identified for the HM1 membranes loaded by each method. The membranes loaded by diffusion presented an API release comprising an initial fast release (burst phase) followed by a lower release state. Figure 5A shows that for both APIs, the burst period occurred within the first 4 min where around 75% was released. This initial burst is often attributed to the rapid release of surface-associated or weakly bound API molecules [36]. After that period, a slow release was observed, and the release rate reached 100% within 25 min, for both APIs. This indicates that the release mechanism for HM1_D_CAF_ and HM1_D_DS_ membranes is primarily controlled by diffusion. For the HM1_D_DS_ membranes, the release rate was further promoted by the disintegration of the membrane observed during the release experiment. Disintegration might be induced by ion exchange, leading to the leaching of Fe cations from the membrane structure during immersion in a diclofenac solution. This exchange of ions can disrupt the crosslinking bonds within the membrane, leading to a loss of the structural integrity of the membrane. In the end, the loaded membrane showed a fragile structure that disintegrated when immersed in the release medium. Xylan/chitosan hydrogel films loaded following a diffusion mechanism presented a similar release profile of diclofenac in PBS solution [37]. The release profile demonstrated an initial burst phase with 43% of the drug being released within the first 5 min, and the total released after 60 min. Following similar loading conditions, the caffeine-loaded CGC hydrogels also demonstrated an analogous behavior; however, the maximum release was achieved within 3 h of the experiment [20]. Considering that a similar amount of caffeine per hydrogel volume was loaded through the same loading method in CGC hydrogels and HM1 membrane structures (11.98 ± 1.29 and 10.07 ± 1.89 mg/cm^3^), the faster release in the membranes might be essentially explained by the larger surface area of membranes that promoted faster release by providing more sites for caffeine molecules to be released from the hydrogel matrix [38]. Muchová et al. [14] also reported a burst release kinetics of caffeine from dialdehyde cellulose/PVA hydrogel films due to the low thickness of the structures which decreases the caffeine diffusion path. After 8 h, up to 90% of the caffeine was released which might be suitable for topical drug delivery.

As shown in Figure 5B, the release of caffeine and diclofenac sodium from the HM1_M_CAF_ and HM1_M_DS_ membranes, respectively, was extended when compared to HM1_D_CAF_ and HM1_D_DS_ membranes and revealed differences in release profiles. It can be observed that for HM1_M_CAF_ membranes the release occurred following two stages: an initial burst release during the first 16 min, where about 30% of the caffeine was released, followed by a second phase with a slow release of the caffeine. During the last phase, the maximum caffeine released (52.6 ± 4.7%) was achieved after 150 min of the experiment. Likewise, whey protein hydrogels enriched with CaCl_2_ were loaded with caffeine through the mixing method, and an analogous maximum of caffeine was released (50–55%) within 4 h of the experiment [39].

The release profile of diclofenac from HM1_M_DS_ membranes also comprises an initial burst release where 36% of the diclofenac was released in the first 15 min (Figure 5B). After that period, a decrease in the release rate was observed, reaching the maximum cumulative release (100%) after 300 min. This behavior was also obtained for the magnetic PVA/carrageenan nanocomposite hydrogels that exhibited a diclofenac release between 75% and 85% in PBS solution, after 7 h of the experiment [40].

The difference between both release profiles can be justified by the way that APIs were incorporated into the hydrogel matrix, which affected their distribution and subsequent release behavior. In fact, in the diffusion method, the API can be easily deposited in microporous spaces of the hydrogel, and drug molecules adhere to the surface of the hydrogel, leading to faster release rates due to the immediate availability of the drug. When the API is loaded by mixing, their molecules are physically or chemically entrapped within the hydrogel matrix, providing a more controlled and sustained release since diffusion through the hydrogel network is needed [29].

Since the release of both APIs from the membranes is controlled mainly by diffusion [41], the first 60% of the release data were modeled according to the Korsmeyer–Peppas model [42]. Based on the regression coefficients (R^2^), all the release data fitted in the model since the R^2^ values were higher than 0.97 (Table 3).

As listed in Table 3, the kinetic parameters revealed that all the HM1 membranes follow a simple Fickian diffusion with n values below 0.45. These results indicate that until 60% of the release, the physicochemical properties of the API used had no significant impact on the release mechanism. Moreover, these n values suggest that the drug release mechanism is purely controlled by diffusion, with the drug being diffused faster through the membrane matrix than the process of polymeric chain relaxation [43]. It was demonstrated that following an identical loading method, both APIs presented similar n values. Using the diffusion method, HM1_D_CAF_, and HM1_D_DS_ membranes showed n values of 0.392 and 0.322, respectively, while using the mixing method HM1_M_CAF_, and HM1_M_DS_ obtained n values of 0.246 and 0.233, respectively. Several other structures have been reported to have similar release mechanisms for caffeine and diclofenac sodium. For example, starch-based hydrogels containing acrylamide and prepared by diffusion method also revealed a caffeine release rate dominated by Fickian diffusion (*n* = 0.17), determined by the interactions between caffeine and the monomers [44]. Similar n values (0.3–0.35) and consequent release mechanisms were obtained for caffeine release from PVA/chitosan hydrogels prepared by the mixing method [22]. Qiao et al. [45] also reported a mechanism controlled by Fickian diffusion (*n* = 0.27) for diclofenac sodium release from gelatin-polyacrylamide hydrogels loaded by diffusion.

## 3. Materials and Methods

### 3.1. Materials

FucoPol composed of 36%mol of fucose, 33%mol of glucose, 26%mol of galactose, and 5%mol of glucuronic acid, with a total acyl group content of 7.8 wt.%, was obtained from the cultivation of the bacterium *Enterobacter* A47, as described by Concórdio-Reis et al. [46]. The polymer presented a protein content of 14.3% and an inorganic salt content of 1.4%. It has a molecular weight of 3.19 × 10^6^ Da, with a polydispersity index of 1.90. Caffeine (99%) and diclofenac sodium salt (98%) were purchased from Alfa Aesar and Tokyo Chemical Industry Co., respectively.

### 3.2. Preparation of HM1 Membranes

The HM1 membranes were fabricated as described in Araújo et al. [18]. Briefly, a FucoPol solution (1 wt.%) was cast into a cylindrical silicone (50 mm diameter, 3 mm height) mold and immersed into an aqueous FeCl_3_ solution (1.5 g/L of Fe^3+^, 250 mL), at room temperature, for 2 h. After a washing step with deionized water (250 mL, 1 h), the HMs were removed from the mold and cut with a stainless-steel cylindrical mold (25 mm diameter) to promote better handling. Then, an additional washing step with deionized water (250 mL) was performed under continuous stirring (150 rpm) until constant conductivity (~1 µS/cm) was achieved. Finally, the HM1 membranes were freeze-dried (−98 °C, 0.03 mbar) and the obtained structures were stored in a closed vessel, at room temperature, until further use.

### 3.3. Loading of the HM1 Membranes with APIs

The HM1 membranes were individually loaded with each of the APIs, namely caffeine (99%, Alfa Aesar) or diclofenac sodium salt (98%, Tokyo Chemical Industry Co., Tokyo, Japan) through two different methods: diffusion and mixing. For the APIs loading by diffusion, the pre-weighed freeze-dried HM1 membranes were immersed in caffeine (1.0 wt.%) or diclofenac sodium (0.1 wt.%) solutions, at room temperature, for 24 h. After that period, the loaded HMs were removed from the API solutions, the excess solution was removed by blotting with filter paper, and the samples were freeze-dried. The obtained HM1 membranes loaded with caffeine and diclofenac sodium were labeled as HM1_D_CAF_ and HM1_D_DS_, respectively. The API loading by the mixing method was performed by mixing 2.5 mg of the API powder with the freeze-dried FucoPol sample (100 mg) and dissolving the mixture in deionized water (10 mL), under continuous magnetic stirring (800 rpm), at room temperature. Then, the solution was cast into the silicone mold, and gelation was promoted by immersion in an aqueous FeCl_3_ solution (1.5 g/L of Fe^3+^), for 2 h. Afterward, the API-loaded membranes were washed by replacing the FeCl_3_ solution with deionized water. Then, the HMs were removed from the mold, cut with the cylindrical mold (25 mm diameter), and washed again with deionized water (250 mL, 150 rpm). Finally, the obtained API-loaded membranes were freeze-dried. The obtained HM1 membranes loaded with caffeine and diclofenac sodium were labeled as HM1_M_CAF_ and HM1_M_DS_, respectively.

For quantification of the API content (mg), API-loaded membrane samples were suspended in PBS solution, pH 7.4, at room temperature, for 24 h, for hydrogel disintegration [47]. After complete hydrogel dissolution and subsequent API extraction, the solution was filtered (0.2 µm filters, Whatman, Maidstone, UK), and the concentration of caffeine and diclofenac was determined by measuring the solution absorbance (UV-Vis spectrophotometer CamSpec M509 T, Leeds, UK), at 273 and 275 nm, respectively [13,22]. The drug loading (DL, mg/g) and the entrapment efficiency (EE, %) were calculated using the following equations [10,22]:

DL = API content in the HMs/W_L_, (1)

EE = API content in the HMs/W_API_, (2)

where W_L_ (g) represents the mass of the loaded freeze-dried HM, and W_API_ (mg) represents the mass of API available.

### 3.4. Characterization of the Loaded Hydrogel Membranes

#### 3.4.1. Fourier Transform Infrared (FT-IR) Spectroscopy

The HM1 hydrogel membranes loaded with caffeine and diclofenac sodium were characterized by FT-IR a spectrum two spectrometer (PerkinElmer, Waltham, MA, USA) with a spectra resolution of 0.5 cm^−1^ equipped with the attenuated total reflectance (ATR) accessory. The spectra were recovered at room temperature based on five scans with a wavelength range from 4000 to 400 cm^−1^.

#### 3.4.2. Mechanical Properties

The compressive mechanical properties of the loaded FucoPol HMs were evaluated with a texture analyzer TMS-Pro (Food Technology Corporation, Slinfold, West Sussex, UK), equipped with a 250 N load cell. Samples of loaded HMs with a thickness of 2–3 mm were cut into a cylindrical shape (25 mm diameter) and were compressed up to 50% strain of the original height at a speed rate of 60 mm/s using a plunger (37 mm diameter). The hardness value (kPa) was established as the maximum tension of the compression, and the toughness (kJ/m^3^) corresponds to the area underneath the stress–strain curve of each sample. The compressive modulus (kPa) was obtained as the slope of the initial linear region. All the experiments were performed at room temperature.

#### 3.4.3. Rheological Properties

A modular compact rheometer (MCR92, Anton Paar, Graz, Austria), equipped with a parallel plate geometry (20 mm diameter) with a 1 mm gap, was used to assess the rheological properties of loaded FucoPol HMs. The samples (25 mm in diameter, ~2 mm in thickness) were equilibrated at 25 °C, for 5 min, and the viscoelastic properties were measured by applying frequency sweeps at a constant tension within the linear viscoelastic region for a frequency range between 0.01 and 10 Hz.

### 3.5. In Vitro Release Studies

Drug release studies were carried out by immersing the freeze-dried API-loaded hydrogel membranes in deionized water (10 mL), at 37 °C, under constant stirring (100 rpm). At predetermined periods, 1 mL samples were withdrawn from the receptor medium, and the same volume was replaced with fresh and preheated (37 °C) deionized water. The concentration of caffeine and diclofenac in the withdrawn samples was determined by measuring their absorbance as described above. The APIs cumulative release values were fitted to the Korsmeyer–Peppas model, according to Equation [42]:

M_t_/M_∞_ = *k*t*^n^* (3)

where Mt and M_∞_ represent the amount of API (g) released at time t and infinite time, respectively; k is the kinetic constant characteristic of the drug-polymer interaction; and n is an empirical parameter for the release mechanism. According to this model, for cylinder-shaped samples, when *n* ≤ 0.45 the diffusion mechanism follows the Fickian diffusion (controlled diffusion), 0.45 < *n* < 0.89 is characteristic of a non-Fickian diffusion (anomalous transport), and *n* ≥ 0.89 represents a relaxation-controlled diffusion (controlled swelling) [42].

## 4. Conclusions

In this study, FucoPol HMs were successfully loaded with caffeine and diclofenac sodium as model APIs, following two loading methods: diffusion and mixing. The membranes demonstrated the ability to be loaded with both APIs by either method, and the presence of the APIs in the membrane structures was confirmed by FT-IR analysis. However, the incorporation of diclofenac sodium by diffusion led to the leaching of the crosslinking agent, and the membrane structure suffered some physical alterations, including changes in the macroscopic appearance, and a decrease in the mechanical and rheological parameters. Except for this structure, all developed membranes displayed improved mechanical and rheological properties when compared to the non-loaded ones. For both APIs, a high release rate was obtained for the membranes loaded by diffusion, whereas sustained and extended release was observed for those prepared by the mixing method. Moreover, all loaded membranes displayed a release profile following a Fickian diffusion, suggesting that the initial release phase occurred independently of the API used. The findings of this study highlight the potential of the developed FucoPol HMs to be used as drug-delivery systems. Nevertheless, future studies will be undertaken focused on exploring additional formulation parameters and optimization strategies to further enhance the entrapment efficiency and release performance of the HMs for different APIs. Furthermore, in vitro drug permeation studies using skin models will be conducted, as well as the evaluation of the biocompatibility and pharmacology of the drug-loaded HMs. Such studies will validate the use of FucoPol HMs as novel and efficient drug-delivery systems.

## Figures and Tables

**Table 1:** APIs concentrations in the loading solutions, drug loading (DL) capacity, entrapment efficiency (EE), and API content in the HM1 membranes.

API	Caffeine		Diclofenac Sodium	
Loading Method	Diffusion	Mixing	Diffusion	Mixing
Loading solution (wt.%)	1.0	-	0.1	-
API concentration (wt.%)	-	0.025	-	0.025
DL (mg API/g dry gel)	101.9 ± 19.1	78.6 ± 2.5	53.9 ± 11.8	82.3 ± 5.1
EE (%)	0.6 ± 0.1	25.8 ± 3.5	1.6 ± 0.2	25.4 ± 3.6

**Table 2:** Mechanical properties (under 90% strain) and storage (G′) and loss (G″) moduli (measured at 1 Hz) of the HM1 membranes loaded with caffeine (HM1_D_CAF_ and HM1_M_CAF_) or diclofenac sodium (HM1_D_DS_ and HM1_M_DS_), compared to the non-loaded membranes (HM1).

Sample ID	Mechanical Properties			Rheological Properties	
	Hardness (kPa)	Compressive Modulus (kPa)	Toughness (kJ/m^3^)	G′ (Pa)	G″ (Pa)
HM1	32.4 ± 5.8	56.3 ± 7.8	1.4 ± 0.1	285.8 ± 36.1	36.7 ± 5.5
HM1_D_CAF_	130.0 ± 5.3	34.1 ± 5.2	41.2 ± 0.6	1014.9 ± 109.7	113.2 ± 13.7
HM1_M_CAF_	60.4 ± 7.2	66.5 ± 8.5	16.3 ± 0.5	609.7 ± 78.3	80.3 ± 2.7
HM1_D_DS_	7.3 ± 1.2	4.6 ± 1.3	1.7 ± 0.4	19.8 ± 3.8	2.2 ± 0.1
HM1_M_DS_	81.8 ± 3.4	67.7 ± 2.5	19.7 ± 0.6	421.0 ± 107.3	40.6 ± 10.2

**Table 3:** Korsmeyer–Peppas model parameters obtained from the in vitro release kinetics of HM1 membranes; R^2^, regression coefficient; *n*, release exponent.

Sample ID	Loading Method	*n*	R^2^
HM1_D_CAF_	Diffusion	0.392	0.994
HM1_D_DS_	Diffusion	0.322	0.995
HM1_M_CAF_	Mixing	0.246	0.978
HM1_M_DS_	Mixing	0.233	0.971

## Data Availability

Data will be made available upon request.
